# Development and immunological evaluation of an mRNA-based vaccine targeting *Naegleria fowleri* for the treatment of primary amoebic meningoencephalitis

**DOI:** 10.1038/s41598-023-51127-8

**Published:** 2024-01-08

**Authors:** Muhammad Naveed, Urooj Ali, Tariq Aziz, Khizra Jabeen, Muhammad Hammad Arif, Metab Alharbi, Abdullah F. Alasmari, Thamer H. Albekairi

**Affiliations:** 1 Department of Biotechnology, Faculty of Science and Technology, University of Central Punjab, Lahore, 54590 Punjab Pakistan; 2 Department of Biotechnology, Quaid-I-Azam University Islamabad, Islamabad, 45320 Pakistan; 3 Laboratory of Animal Health, Food Hygiene and Quality, Department of Agriculture, University of Ioannina, 47132 Arta, Greece; 4 Department of Pharmacology and Toxicology, College of Pharmacy, King Saud University, Post Box 2455, 11451 Riyadh, Saudi Arabia

**Keywords:** Biochemistry, Computational biology and bioinformatics, Genetics, Immunology

## Abstract

More than 95% of patients fall victim to primary amoebic meningoencephalitis (PAM), a fatal disease attacking the central nervous system. *Naegleria fowleri*, a brain-eating microorganism, is PAM's most well-known pathogenic ameboflagellate. Despite the use of antibiotics, the fatality rate continues to rise as no clinical trials have been conducted against this disease. To address this, we mined the UniProt database for pathogenic proteins and selected assumed epitopes to create an mRNA-based vaccine. We identified thirty B-cell and T-cell epitopes for the vaccine candidate. These epitopes, secretion boosters, subcellular trafficking structures, and linkers were used to construct the vaccine candidate. Through predictive modeling and confirmation via the Ramachandran plot (with a quality factor of 92.22), we assessed secondary and 3D structures. The adjuvant RpfE was incorporated to enhance the vaccine construct's immunogenicity (GRAVY index: 0.394, instability index: 38.99, antigenicity: 0.8). The theoretical model of immunological simulations indicated favorable responses from both innate and adaptive immune cells, with memory cells expected to remain active for up to 350 days post-vaccination, while the antigen was eliminated from the body within 24 h. Notably, strong interactions were observed between the vaccine construct and TLR-4 (− 11.9 kcal/mol) and TLR-3 (− 18.2 kcal/mol).

## Introduction

*Naegleria fowleri* (*N. fowleri*), a eukaryotic unicellular amoeba, belongs to Percolozoa and is the most lethal water-borne amoeba, with a 97% mortality rate since its emergence. *N. fowleri* has been associated with brain-eating infections causing brain inflammation and meningoencephalitis^1^. Meningitis is the membrane cerebrum of the brain digested by the *N. fowleri* and leads to brain swelling called encephalitis, a fatal condition. *N. fowleri* is a natural thermophile found in humid air but prominent niches in fresh waters, untreated swimming pools, thermal wells, lakes, and rivers^2^. A study claimed that this microorganism is present in all continents except Antarctica and epidemically occurred in U.S., India, and Pakistan as a silent killer^3^. *N. fowleri* enters the body when tainted water travels up the nose, as when swimming or nasal irrigation is used. Amebae traverses the cribriform plate by passing through the olfactory tract directly into the brain, initiating brain-eating infection, also known as meningoencephalitis. Patients experience headaches, fever, nausea, stiff neck, disorientation, convulsions, and delusions after a few days of being exposed^4^. *N. fowleri* pathogenicity and the strong immunological reaction brought on by its presence cause severe nerve injury and consequent CNS tissue destruction, frequently leading to death. The illness advances quickly and typically results in death 10 days after exposure. The median period from the beginning of symptoms to death in PAM cases is 5 days, and the case fatality rate is above 97%. However, consuming polluted water does not spread the disease^5^.

In modern times, numerous occurrences of *N. fowleri* infections have been documented in various regions across the globe, eliciting apprehension among public health authorities and communities. In 2022, the United States experienced a worrisome occurrence characterized by identifying three verified instances of *N. fowleri* infection^6^. These were documented in individuals hailing from Iowa, Nebraska, and Arizona and ascribed to their contact with fresh water. In light of this emerging public health concern, a novel therapeutic approach for treating *N. fowleri* infection, namely miltefosine, has exhibited encouraging effectiveness in controlled laboratory environments^7^. It is worth mentioning that miltefosine has already been approved as a pharmaceutical intervention for treating *Leishmania*, responsible for a different type of parasitic infection^8^. In addition, there have been reported instances of *N. fowleri* infection in individuals who employed tap water for sinus rinsing using net pots. This observation suggests a possible transmission mode and underscores the significance of exercising prudence when utilizing neti pots, especially in areas where *N. fowleri* is known to exist.

In March 2023, the Florida Department of Health reported an infection of *N. fowleri* in Charlotte County^9^. The department opted not to reveal the identity of the individual who tested positive for the infection, nor did they provide any additional information regarding the individual's condition^9^. Nevertheless, there has been a suggestion that the infection could potentially be attributed to the utilization of tap water in sinus rinse practices^5^. The contraction of *N. fowleri* presents a significant hazard, resulting in the development of primary amebic meningoencephalitis (PAM), a severe brain infection.

On May 31, 2023, Karachi encountered a lamentable epidemic of three fatalities associated with *N. fowleri* infections^10^. Concerns have been expressed regarding the potential existence of unreported cases, suggesting that the number of affected individuals may be greater^10^. The occurrence brought attention to a fundamental problem: a significant segment of the populace faced a dearth of access to hygienic and uncontaminated water sources. Consequently, individuals were compelled to ingest water that had been contaminated, rendering them susceptible to a range of waterborne ailments such as Typhoid, Gastroenteritis, Hepatitis A and E, and Cholera. The contaminated water also acted as a medium for the *N. fowleri* amoeba, which can potentially infiltrate the brain via the nasal passages, resulting in significant neurological impairment. In the Alappuzha district of Kerala, a regrettable event occurred in July 2023, wherein a 15-year-old male individual succumbed to an uncommon infection caused by *N. fowleri*^11^. The investigation unveiled that the probable origin of the amoeba was a nearby stream, which the individual had previously utilized for bathing purposes. On July 4, 2023, Lahore experienced a notable public health incident whereby it recorded its inaugural fatality linked to *N. fowleri*, a parasitic amoeba known for its ability to cause brain infections^12^. This case represents a significant development, as the amoeba in question had previously been linked to fatalities in different geographical areas. As a result, it has garnered attention and increased awareness regarding the potential dangers associated with this pathogen.

The Center for Disease Control and Prevention (CDC) revised its guidelines for preventing *N. fowleri* infection in 2019^13^. The updated guidelines recommend that individuals refrain from swimming in warm freshwater, particularly in periods of high temperatures, due to the favorable conditions that facilitate the rapid growth and spread of the amoeba^14^. Moreover, it is recommended that individuals rinse their nasal passages with bottled water following freshwater swimming to minimize the potential colonization of *N. fowleri*^13^. Implementing precautionary measures is of utmost importance in reducing the occurrence and consequences of *N. fowleri* infections and protecting the general population's well-being in areas susceptible to this potentially fatal amoeba. The therapeutic approaches targeting *N. fowleri* demonstrate encouraging progress in addressing this lethal pathogen. Utilizing in-silico analysis, specifically identifying potential drug targets, offers a valuable initial step in pursuing drug discovery endeavors. For example, Saleem et al.^15^ identifying two proteins rich in glutamine from the recently discovered Karachi-NF001 strain presents a promising opportunity for developing efficacious pharmaceutical interventions. Regarding the development of vaccines, there has been promising advancement in constructing a multi-epitope vaccine construct. By activating both B and T lymphocytes, this vaccine candidate can elicit a vigorous immune response against *N. fowleri*^15^. They incorporated linkers and an adjuvant to augment its efficacy, thereby providing promise for the implementation of efficacious preventive measures.

In addition, Gutiérrez-Sánchez et al.^16^ research efforts examining immunoprotective responses have identified two peptide-based vaccine antigens promising candidates for preventing meningitis induced by *N. fowleri*. The immunogenic potential of these antigens is indicated by the substantial protection they provide and the increased levels of IgA, IgG, and IgM in both serum and nasal wash samples^16^. Moreover, the potential therapeutic efficacy of cathepsin B as a target is promising. The enhancement of survival rates in mice infected with *N. fowleri* through the inhibition of cathepsin B^17^ underscores the importance of this enzyme in *N. fowleri* infections. It highlights its potential as a viable target for developing therapeutic interventions. Consequently, a vaccine with long-term safety and effectiveness is the best option to develop immunity against the disease before you get infected with the suspected disease^18,19^. This work is based on a key purpose to develop a novel mRNA-based vaccination against *N. fowleri*. Although, to the best of our knowledge, an mRNA-based vaccine against *N. fowleri* has not been reported yet, these candidates have amazing potential against pathogenic infections. mRNA vaccines offer rapid development potential against evolving pathogens, like COVID-19. They trigger immune responses without using live viruses, ensuring safety. Their adaptable platform enables quick modifications for new variants. Additionally, they do not integrate into the genome, posing no risk of altering genetic information. In this work, immunogenic, harmless, and non-allergen epitopes for B and T immune cells are discovered using online available tools. The potential for autoimmunity to develop in response to the chosen epitopes is also assessed. The vaccine candidate's structure is predicted and docking with host immune molecules (TLR-3 and TLR-4) is carried out. Finally, immune simulation and molecular dynamic modeling have been used to verify the vaccination complex's stability.

## Materials and methods

### Protein sequence retrieval

The whole available proteome of *Naegleria fowleri* was acquired from UniProt, https://www.uniprot.org/proteomes/. Pathogenic proteins in FASTA format were downloaded to predict effective epitopes. Only five pathogenic but non-allergenic and non-toxic proteins were selected for vaccine construction from the *N. fowleri* proteome, shown in Table 1. Such proteins and peptides are crucial in vaccines to prevent adverse reactions and ensure overall safety for recipients.

**Table 1:** Selected pathogenic proteins from the whole available proteome of *N. fowleri.*

Sr	Protein name	Accession number
1	Phosphate transporter	A0A6A5C2W9
2	Methionine aminopeptidase 2	A0A6A5BP86
3	Phospholipid-transporting ATPase	A0A6A5BY12
4	Alpha-1, 3/1, 6-mannosyltransferase ALG2	A0A6A5BFM1
5	Putative Naegleria-specific protein	M1H4N2

### B-cell epitopes analysis

ABCpred, accessed at https://webs.iiitd.edu.in/raghava/abcpred/ABC_submission.html, an online web server, was run for the B-cell epitopes. The shortlisted proteins were delivered with an epitope length criterion of 16-mer and threshold of 0.51. The top ten epitopes were selected from five proteins to derive the vaccine candidate.

### T-cell epitopes analysis

The computational techniques of the IEDB server for MHC-I http://tools.iedb.org/mhci/ were run to screen CTL epitopes. The server required the selected proteins' FASTA sequences to run. The MHC-I IEDB server method known as NetMHCpan BA 4.1 produces numerical estimates of the affinities of any peptide-MHC class I interaction^20^. 9 and 10-mer lengths were used to determine the epitopes. Human full-set HLA was utilized for the epitopes' final prediction findings and sorted by IC evaluation. The Helper T-Lymphocytes epitopes were predicted under the MHC-II IEDB server NetMHCpan BA 4.1 module http://tools.iedb.org/mhcii/help/ by selecting full set human allele with the default values.

### Epitopes immunogenicity estimation

The antigenicity of immune cell epitopes was predicted utilizing Vaxijen2.0^21^, an online available tool having a threshold value of 0.5 http://www.ddg-pharmfac.net/vaxijen/VaxiJen/VaxiJen.html, was run to check the immunogenicity of designed immune cells epitopes. Similarly, allergenicity was predicted by operating AllerTOP v. 2.0 https://www.ddg-pharmfac.net/AllerTOP/^22^. ToxinPred^23^, used freely at https://webs.iiitd.edu.in/raghava/toxinpred/, was applied to check whether the designed epitopes were toxic or harmless.

### Homology analysis

The NCBI BLASTp database https://blast.ncbi.nlm.nih.gov/Blast.cgi?PAGE=Proteins^24^ was examined for the predicted peptides to exclude any potential autoimmunity. All the remaining peptides were then considered potential non-homologous if their E value was larger than 0.05 and utilized in vaccine construction.

### mRNA-based vaccine construct

Designed epitopes from pathogenic microorganism proteins were linked by EAAAK, AAY, GPGPG, and KK sequences, enabling the autonomy of epitopes. RpfE was employed as an immune-stimulating adjuvant. The mRNA vaccination must have a 5′ UTR-Kozak sequence along with a tPA Signal peptide and MITD sequence, followed by the Stop codon-3′ UTR-Poly (A) tail^25^.

### Assessment of physicochemical qualities of the candidate

The physicochemical properties of the vaccine are expected by operating VaxiJen to determine antigenicity, AllerTOPv 2.0 server for allergenicity. The PROTPARAM website https://web.expasy.org/protparam/^26^, which offers information on vaccine properties such as composition, molecular weight, projected pI, and GRAVY, was utilized to determine physiochemical parameters.

### Predicting the structure

The 2D structure of constructed sequence was predicted by the PsiPred online tool http://bioinf.cs.ucl.ac.uk/psipred/^27^. Here we got the percentage of coil, alpha helix, beta sheets, and turn involved in the final structure. The tertiary structure of the protein was produced with trRosetta, found freely at https://yanglab.nankai.edu.cn/trRosetta/^28^ that utilizes energy minimizations and model constraints to derive the 3D structure. The Procheck, ERRAT, and Ramachandran Plot, at the saves website of UCLA https://saves.mbi.ucla.edu/^29^ were utilized to verify the proteins' estimated structural analyses. The plot demonstrated stability, effectiveness, torsional angles, and the presence of amino acids in different regions of the vaccine structure.

### Molecular docking of the candidate with immune molecules

Molecular modeling/docking assessed the vaccine construct's molecular interface and immune response activation potential. The construct was docked with TLR-3 (PDB ID: 1ZIW) and TLR-4 (PDB ID: 3FXI) using the ClusPro server^30^, available at https://cluspro.bu.edu/login.php, to analyze their interaction between them and complexes with the lowest energy was downloaded. We utilized these two receptors because they recognize parasitic molecular patterns in the human body. So, if a parasite attacks a human host, these will be the first line of response and defense against it, that is why the vaccine candidate must elicit a stimulation of these receptors.

#### Immune simulation analysis

C-Immsim (https://150.146.2.1/C-IMMSIM/index.php), a free online immune simulation webserver^31^, was implemented to assess the antibody responses of the vaccine design. The immunological response was imitated using a process centered on the interaction of epitopes with lymphocyte receptors.

#### In-silico expression analysis

The peptide sequence of vaccine design was optimized for efficient synthesis within human cells. Jcat tool http://www.jcat.de/Literature.jsp for codon Optimization was employed^32^, whereby the codon adaptation index (CAI) helped define the effectiveness of mRNA translation. SnapGene was used for the bioinformatic-assisted cloning process.

#### MD simulation analysis

**Figure 1:**
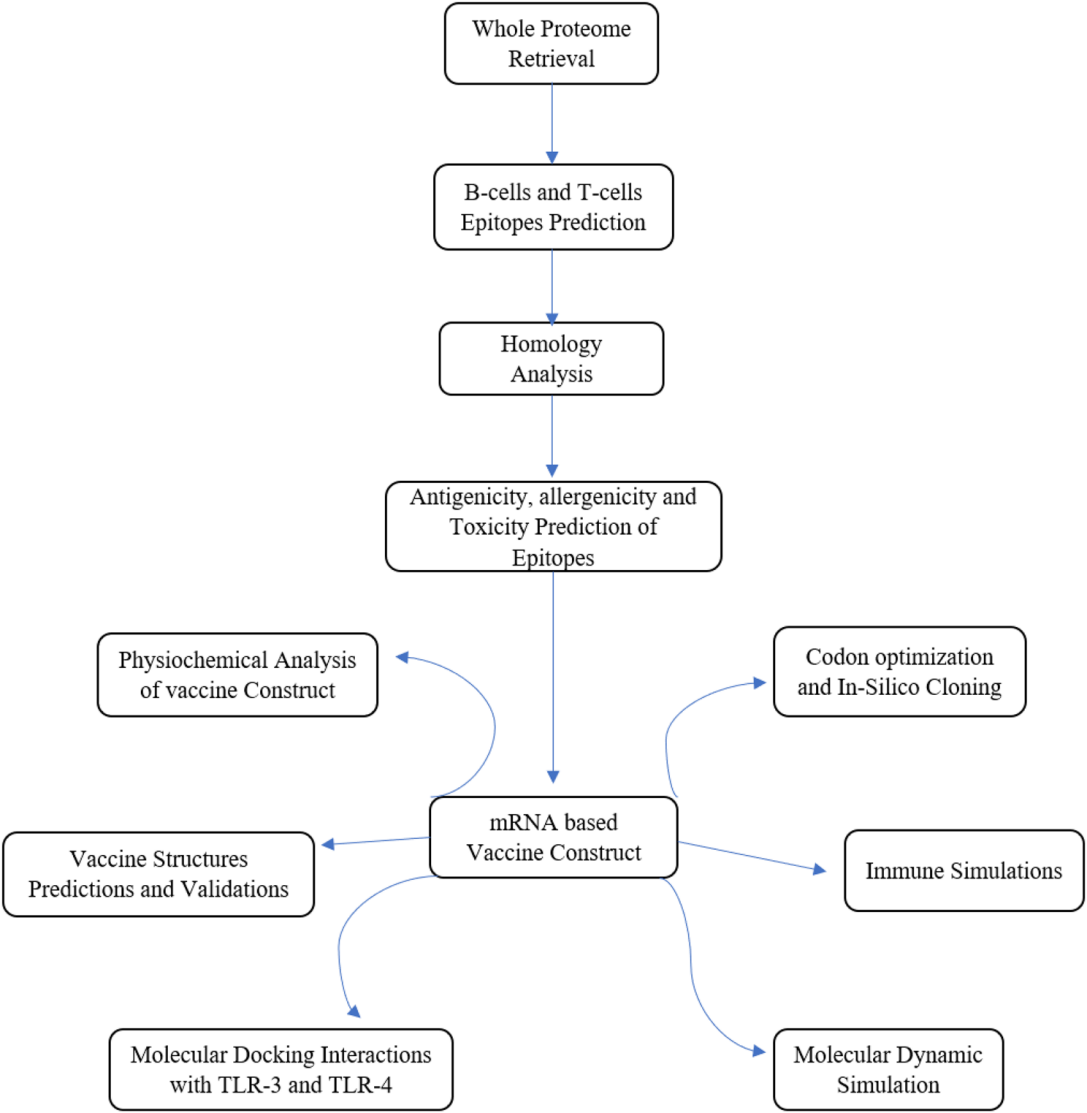
Overview of the adopted methodology to derive the mRNA-based vaccine construct.

The IMODs server https://imods.iqfr.csic.es/ was used to confirm the stability of the docked structures^33^. The TLR-3/vaccine and TLR-4/vaccine complexes were run on iMODs server to study the stability and dynamics of these complexes along with the suitable binding energies. A summary of the adopted methodology is provided in Fig. 1.

## Results

### Protein retrieval

The five antigenic proteins with accession number of A0A6A5C2W9 (Phosphate transporter), A0A6A5BP86 (Methionine aminopeptidase 2), A0A6A5BY12 (Phospholipid-transporting ATPase), A0A6A5BFM1 (Alpha-1, 3/1, 6-mannosyltransferase ALG2), and M1H4N2 (Putative Naegleria-specific protein) were retrieved from the UNIRPOT database.

### Epitopes analysis

ABCpred was used to predict B and T lymphocyte-specific epitopes were combined to produce multiple epitope vaccinations. A humoral immune response is an immunological response initiated by B-cells and reliant on antibodies. A total of ten B-cell epitopes of five proteins were predicted, as shown in Table 1. Two epitopes from each protein were used by checking their antigenicity and allergenicity and score on ABCpred. In contrast, both MHC epitopes were anticipated from T-cell epitopes, and this strategy was utilized to predict T-cell epitopes using two different servers of IEDB, both of which utilized NetMHCpan BA 4.1. This study determined ten MHC-I and ten MHC-II epitopes, as depicted in Tables 2, 3 and 4.

**Table 2:** List of B-cell epitopes candidate to design vaccine with predicted allergenicity and antigenicity.

Rank	Epitope	Antigenicity	Allergenicity
1	EMMITTTNTHSNDHDT	1.3435	Non-allergen
	MAMKYASTEEEEEENP	0.9839	Non-allergen
2	GVCSHYMKDFHKKVNP	0.8586	Non-allergen
	DQTKMEENEFFAIETF	1.5383	Non-Allergen
3	TESQKTTTTMVRRDDV	0.7198	Non-Allergen
	SWSIENDTEMRFEKLK	0.6888	Non-Allergen
4	TALYMGTYGEDDFVTS	1.2623	Non-Allergen
	EVDRQDEQRSNGPSNL	0.6077	Non-Allergen
5	HPHNEEETVRNFGNCI	0.6462	Non-Allergen
	LAGGYDPRIAENIQVL	0.8642	Non-Allergen

**Table 3:** List of MHC-I epitope candidates and alleles to design a vaccine candidate.

Protein No	Epitope	Antigenicity	Allergenicity	Alleles
1	ALFSFLKWTV	1.3435	Non-Allergen	HLA-A*32:01, HLA-A*02:01, HLA-A*02:03
	GSLFIMGSPA	0.9839	Non-Allergen	HLA-A*02:03,
2	KTQKARELLK	0.8586	Non-Allergen	HLA-A*30:01, HLA-A*11:01, HLA-A*03:01, HLA-A*31:01,
	YMKDFHKKV	1.5383	Non-Allergen	HLA-A*02:03, HLA-B*08:01, HLA-A*02:01
3	ADFLHYSFY	1.7553	Non-Allergen	HLA-A*30:02
	VLPITFIVLL	1.7377	Non-Allergen	HLA-A*02:01, HLA-A*02:03, HLA-A*02:06
4	LLVKAFAKYV	1.2311	Non-Allergen	HLA-A*02:03, HLA-A*02:01, HLA-A*02:06
	ENFGQRARQR	1.254	Non-Allergen	HLA-A*68:01, HLA-A*33:01
5	HSGMVTNSMK	1.6696	Non-Allergen	HLA-A*68:01, HLA-A*11:01, HLA-A*30:01, HLA-A*03:01
	MLTALYMGTY	1.3296	Non-Allergen	HLA-B*15:01, HLA-A*30:02, HLA-A*26:01, HLA-A*01:01, HLA-A*68:01

**Table 4:** List of MHC-II epitope candidates and alleles to design a vaccine candidate.

Protein no.	Epitope	Antigenicity	Allergenicity	Alleles
1	EEKKKVQQNPSTTSS	0.8516	Non-allergen	HLA-DPA1*01:03, HLA-DPA1*02:01, HLA-DPA1*02:01, HLA-DPA1*01:03, HLA-DPA1*03:01, HLA-DRB1*15:01, HLA-DRB5*01:01,
	AFTMYWDDKFKPLAM	1.1464	Non-allergen	HLA-DPA1*03:01, HLA-DPA1*02:01, HLA-DPA1*01:03, HLA-DPA1*01:03, HLA-DPA1*01:03, HLA-DRB1*07:01, HLA-DPA1*02:01, HLA-DPA1*02:01, HLA-DRB1*09:01, HLA-DRB1*15:01, HLA-DRB1*13:02, HLA-DRB1*04:05, HLA-DRB3*02:02, HLA-DRB1*12:01, HLA-DRB4*01:01, HLA-DQA1*01:02, HLA-DRB1*01:01, HLA-DRB1*08:02, HLA-DQA1*05:01, HLA-DRB1*11:01, HLA-DRB5*01:01,
2	AIFSVYIQGNLQIGA	1.5081	Non-allergen	HLA-DRB1*15:01, HLA-DRB1*13:02, HLA-DRB4*01:01, HLA-DRB1*12:01, HLA-DRB1*04:05, HLA-DRB1*04:01, HLA-DQA1*01:02,
	MCVLLGPTAWLILST	0.8646	Non-allergen	HLA-DPA1*01:03, HLA-DPA1*01:03, HLA-DPA1*03, HLA-DRB1*07:01, HLA-DRB1*09:01, HLA-DPA1*02:01, HLA-DRB1*15:01, HLA-DRB1*01:01
3	FTMYWDDKFKPLAMA	1.2667	Non-allergen	HLA-DPA1*01:03, HLA-DQA1*01:01, HLA-DQA1*05:01, HLA-DRB1*03:01, HLA-DPA1*01:03, HLA-DPA1*03:01, HLA-DRB3*01:01,
	EENEFFAIETFGSTG	0.6557	Non-allergen	HLA-DRB1*04:01, HLA-DPA1*01:03, HLA-DPA1*02:01, HLA-DRB1*04:05, HLA-DPA1*03:01, HLA-DRB1*08:02, HLA-DQA1*01:0, HLA-DPA1*02:01, HLA-DRB1*09:01, HHLA-DQA1*01:02, LA-DRB1*01:01, HLA-DQA1*05:01, HLA-DRB1*11:01, HLA-DRB1*07:01HLA-DRB5*01:01, HLA-DRB1*15:01
4	FEKKKNIALLVKAFA	0.7803	Non-allergen	HLA-DPA1*02:01, HLA-DPA1*02:01, HLA-DRB1*12:01, HLA-DRB3*02:02, HLA-DRB1*13:02, HLA-DRB1*11:01, HLA-DRB1*08, HLA-DQA1*01:02, HLA-DRB4*01:01, HLA-DPA1*03:01, HLA-DPA1*02:01, HLA-DRB5*01:01, HLA-DRB1*15:01, HLA-DPA1*01:03, HLA-DRB1*01:01, HLA-DRB1*07:01, HLA-DRB1*09:01,
	NSDFTKQIFYESFKR	0.9513	Non-allergen	HLA-DPA1*01:03, HLA-DPA1*02:01, HLA-DPA1*02:01, HLA-DPA1*01:03, HLA-DPA1*03:01, HLA-DRB1*15:01, HLA-DRB5*01:01,
5	ILYNRIFQATVLTML	0.9554	Non-allergen	HLA-DPA1*03:01, HLA-DPA1*02:01, HLA-DPA1*01:03, HLA-DPA1*01:03, HLA-DPA1*01:03, HLA-DRB1*07:01, HLA-DPA1*02:01, HLA-DPA1*02:01, HLA-DRB1*09:01, HLA-DRB1*15:01, HLA-DRB1*13:02, HLA-DRB1*04:05, HLA-DRB3*02:02, HLA-DRB1*12:01, HLA-DRB4*01:01, HLA-DQA1*01:02, HLA-DRB1*01:01, HLA-DRB1*08:02, HLA-DQA1*05:01, HLA-DRB1*11:01, HLA-DRB5*01:01,
	PYLIVATKTDLLVDG	1.2209	Non-allergen	HLA-DRB1*04:05, HLA-DRB4*01:01, HLA-DRB1*07:01, HLA-DRB1*04:01, HLA-DRB1*13:02, HLA-DRB1*03:01, HLA-DRB1*12:01, HLA-DRB1*08:02, HLA-DRB1*09:01, HLA-DRB3*01:01, HLA-DRB1*15:01, HLA-DRB1*01:01, HLA-DRB1*11:01, HLA-DRB5*01:01,

### Vaccine construct and analysis

**Figure 2:**
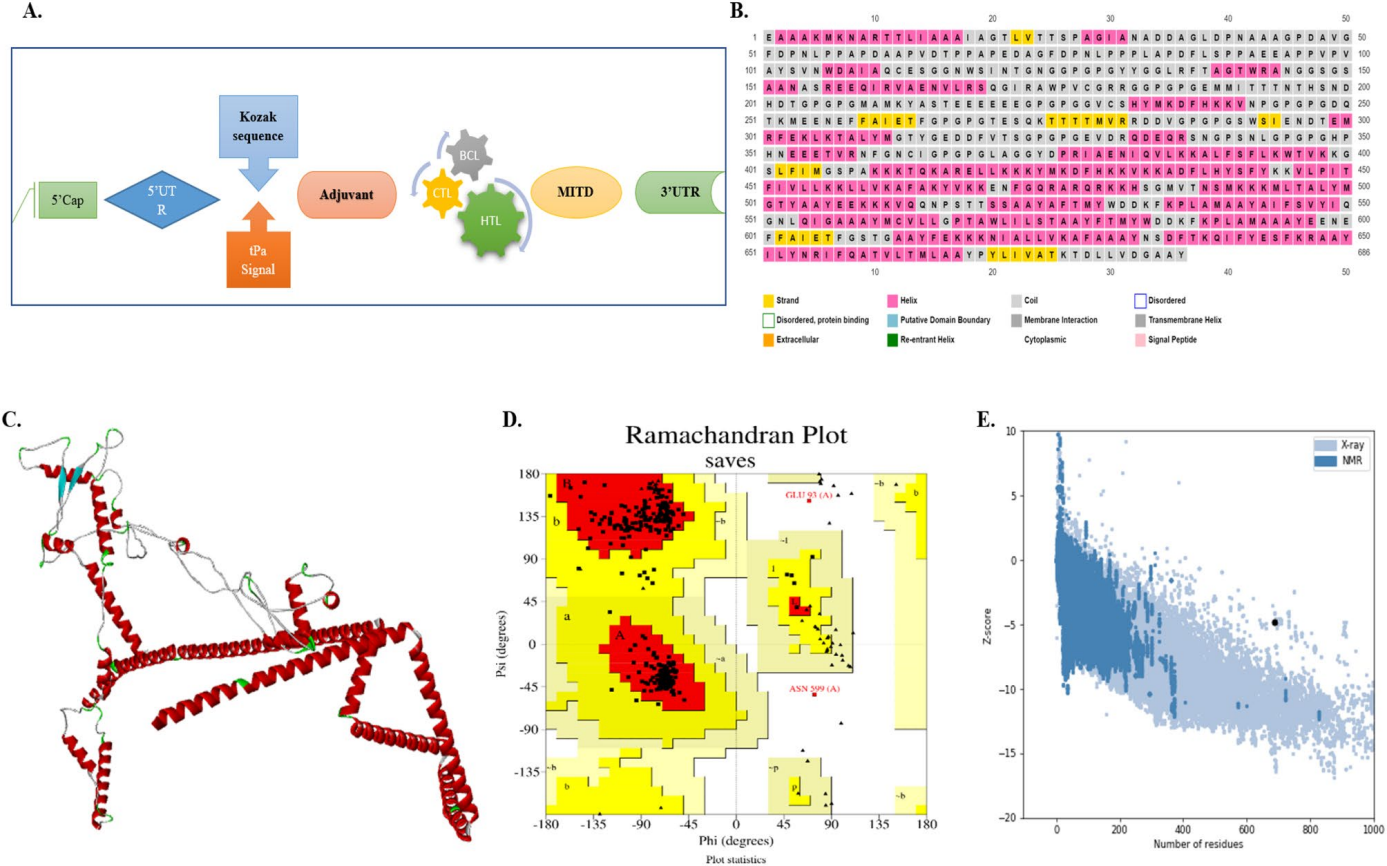
Structural evaluations of the mRNA candidate. (**A**) mRNA construct (**B**) secondary protein structure (**C**) 3D structure anticipated using trRosetta (**D**) Ramachandran plot illustrated amino acid residues in different regions of vaccine construct (**D**) ProSA web run for Z-score evaluation.

The epitopes for the mRNA vaccine candidate were linked using EAAAK, GPGPG, KK, and AAY, along with adjuvants and other sequences, as shown in Fig. 2. The vaccine construct was immunogenic and soluble. Per our evaluation, it will not result in an allergic response or cause any toxicity in the human host. Additionally, Table 5 provides the physiochemical analysis of vaccine design, determined by the ExPasy ProtParam service, evaluating that the construct would be thermally stable. The vaccine candidate was considered hydrophilic according to the GRAVY value of − 0.394.

**Table 5:** Physiochemical properties and analysis of the vaccine construct.

Property	Measurement
Antigenicity	0.8
Allergenicity	Non-allergen
Toxicity	Non-toxic
Formula	C_3347_H_5133_N_881_O_988_S_27_
Total number of atoms	10,376
instability index (II)	38.99
Stable or non-stable	Stable
Aliphatic Index	63.86
Average of hydropathicity (GRAVY)	− 0.394

### Structural prediction

A stable secondary structure was predicted for the mRNA sequence of our vaccine construct. Figure 2B shows that alpha helices comprise the structure's bulk. The 3D model of our candidate, shown in Fig. 2C, was also determined using the TrRosetta server. The PROCHECK service was used to confirm the structure's stereochemical accuracy. Figure 2D's Ramachandran plot displays that 93.8% of residues were in the most desired zones, 5.9% were in the additionally allowed zone, 0.2% were generously allowed, and 0.2% were in the disallowed region. The vaccine has an overall quality factor of 92.2204 checked by using ERRAT. The tertiary protein model was predicted by the ProSA-web server to have a negative Z-score of − 4.78, indicating that it is remarkably compatible in Fig. 2E.

### Immune simulation

**Figure 3:**
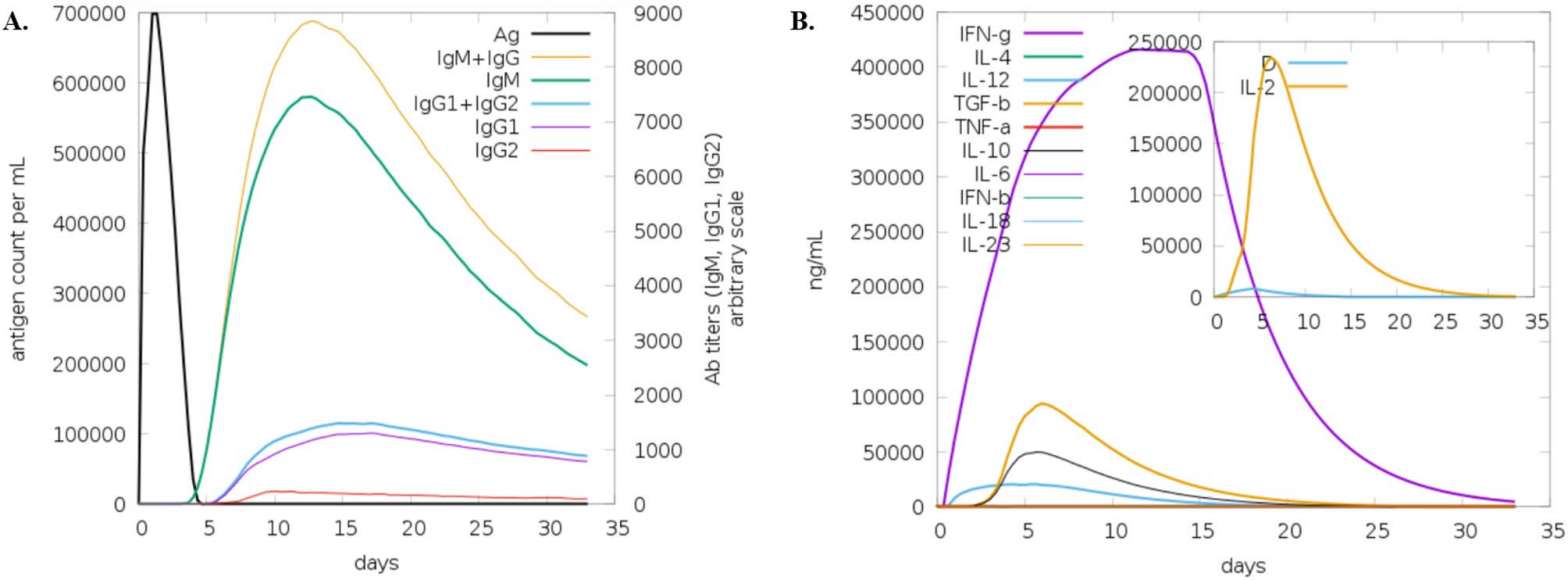
C-ImmSim server applied to check the immune response when the vaccine is injected into the host. Antibody production after vaccine inoculation as antigen (**A**), the stimulation of interferon, interleukins, and tumor necrosis factors (**B**).

We utilized specific MHC class I and II alleles (A0101, B0702, DRB1_0101) to simulate AI-predicted immune responses in a human computational model. Employing these alleles, along with injection details—100 adjuvant units, and 1000 antigen units—enabled us to predict the vaccine's potential impact on the individual's immune system. Immune stimulation revealed that the subsequent responses performed better than the first response. After antigen suppression, immunoglobulin levels were elevated, and it was discovered that IgM was produced in larger amounts than IgG. This increase demonstrated that exposure to antigens led to the development of immunological memory. B-cell isotypes' persistence over time is evidence of memory development in the B-cell population. Additionally, CTL and HTL cells showed an increase in memory formation. The activity of macrophages also increased, although that of dendritic cells stagnated. IFN- and IL-2 levels also increased, as seen in Fig. 3.

### Molecular docking of vaccine construct with TLR-3 and TLR-4

The translated protein model of our mRNA vaccine candidate docked with TLR-3 and TLR-4 human receptors using ClusPro. It provided interpretations with 15 different models along with different binding energy values. In both cases, TLR-3 and TLR-4, the first model was selected with the best minimum binding energy value. Vaccine and TLR-3 complex had a binding energy of − 1293.2 kcal/mol, and TLR-4 had a binding energy value of − 1164.5 kcal/mol.

### Sequence cloning

**Figure 4:**
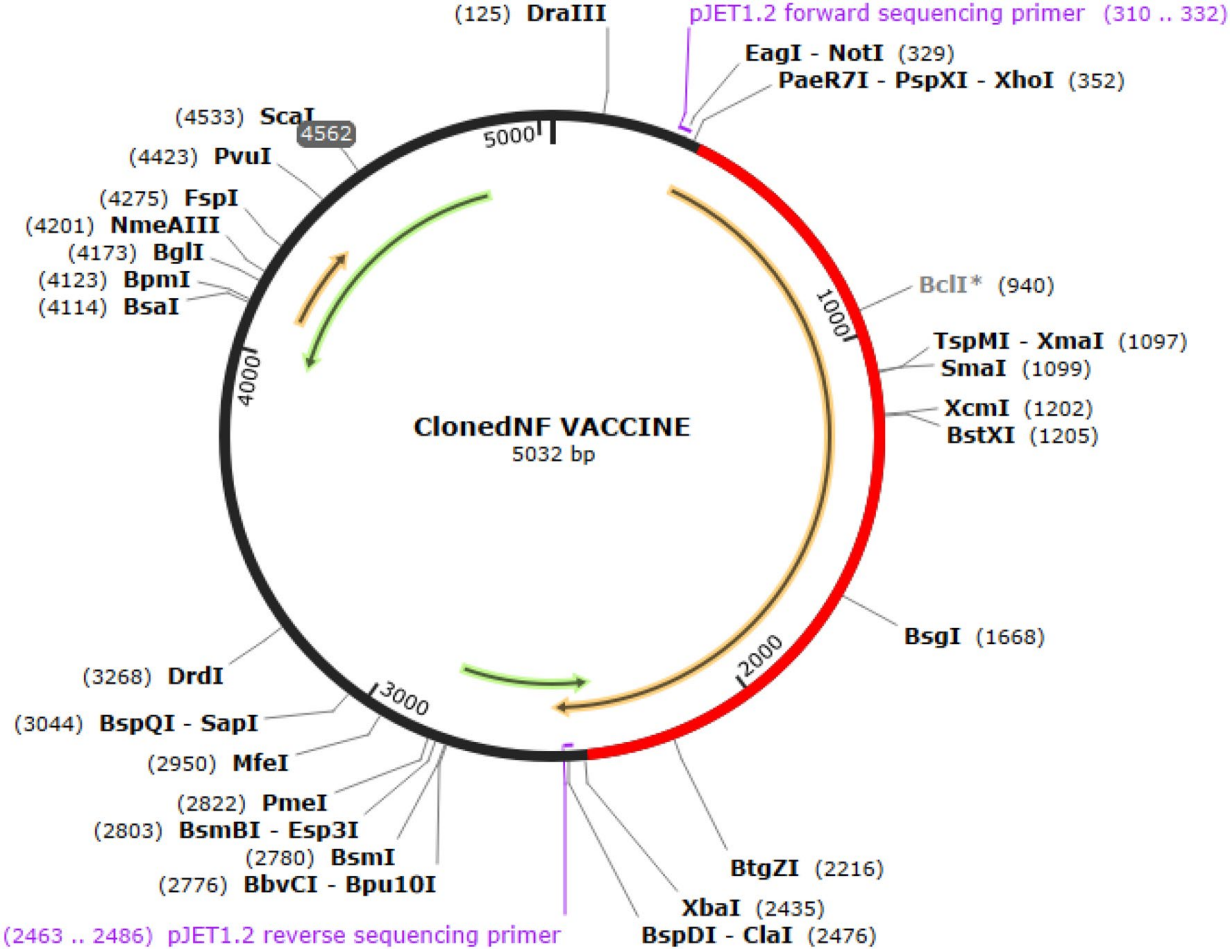
Codon optimization and in-silico cloning of the vaccine construct in the pJET1.2 blunt vector.

The construct sequence was optimized using the codon optimization program Jcat, which resulted in an increased value of CAI 0.9 and a GC content of around 60%. Expression was performed in E. coli, and the optimized DNA sequence and the pJET1.2 blunt-ended vector were used, as shown in Fig. 4.

### Molecular dynamic simulations

**Figure 5:**
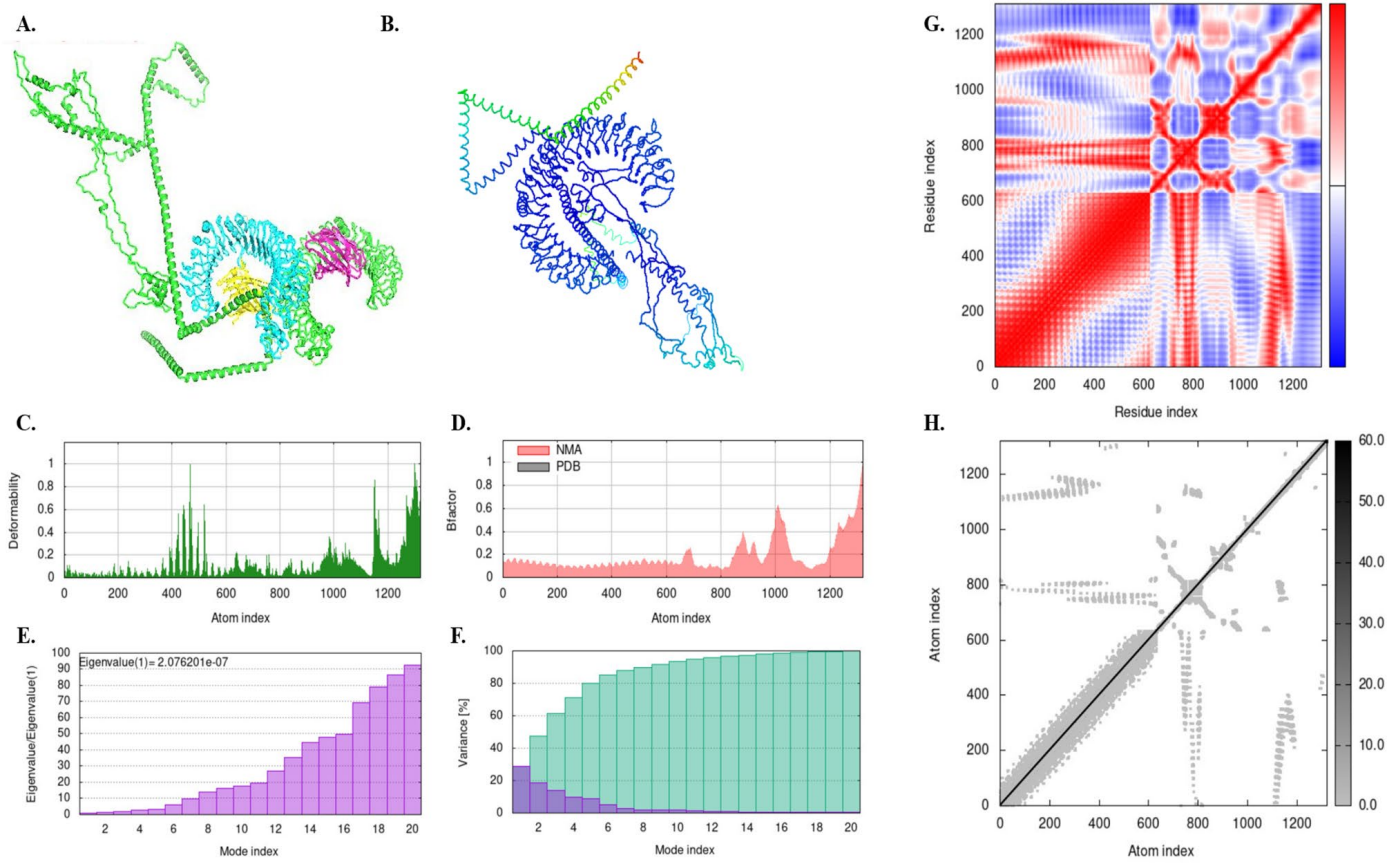
Docking and MD simulations of the vaccine candidate with TLR3; (**A**) the docking complex, (**B**) the complex subjected to NMA mobility, (**C**) The deformability graph, (**D**) the B-factor graph, (**E**) the Eigenvalue plot, (**F**) the variance plot, (**G**) the Co-variance plot, and (**H**) the elastic network map.

**Figure 6:**
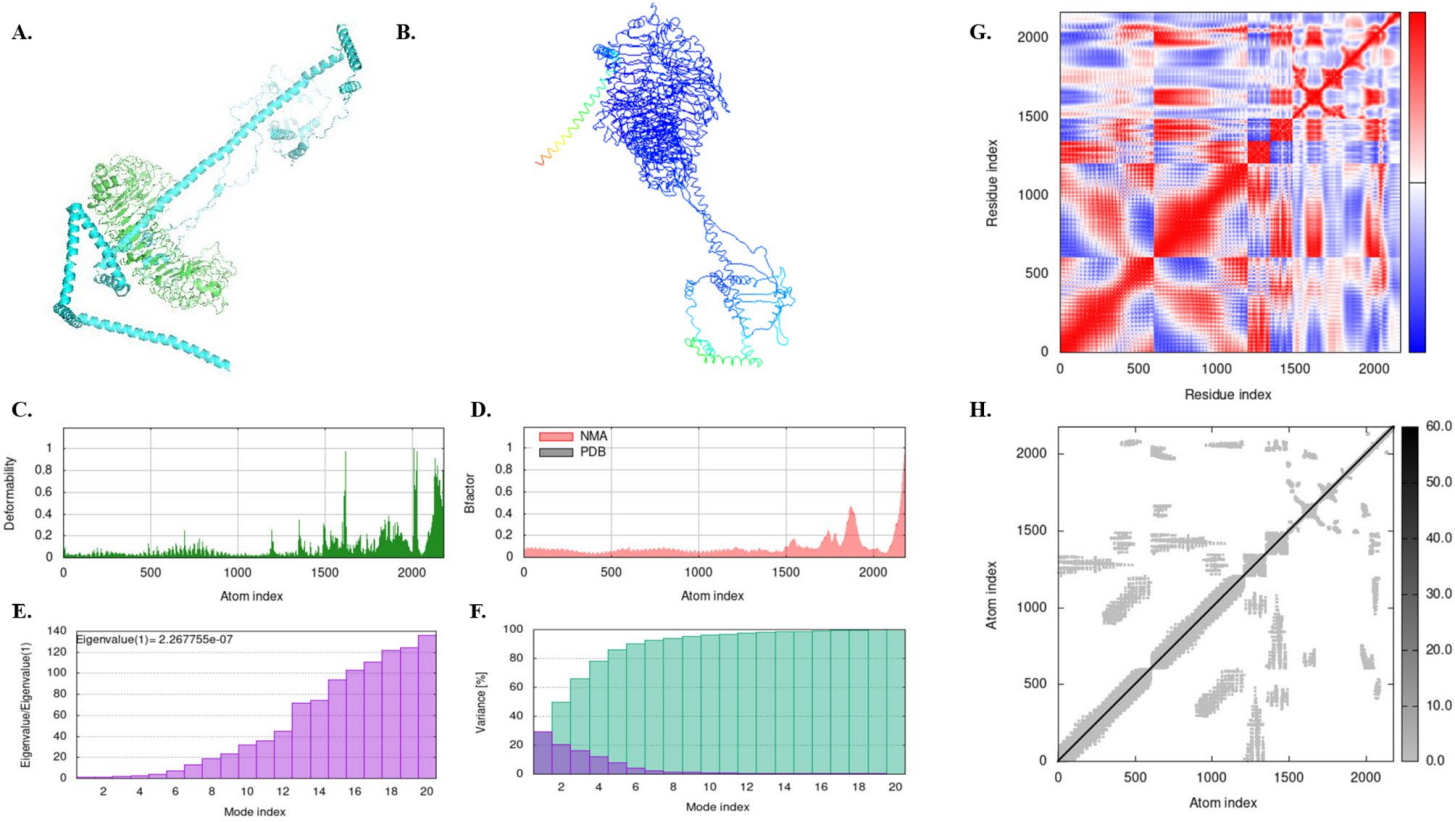
Docking and MD simulations of the vaccine candidate with TLR4; (**A**) the docking complex, (**B**) the complex subjected to NMA mobility, (**C**) the deformability graph, (**D**) the B-factor graph, (**E**) the Eigenvalue plot, (**F**) the variance plot, (**G**) the Co-variance plot, and (**H**) the elastic network map.

The dependability of vaccine construction in the environment was predicted using the B-factor mobility and obtained from molecular dynamic simulation. The complex's B factor, stability, flexibility, residues covariance map, and atom index were analyzed. The MD simulations of both the complexes showed that the relations of vaccine construct with TLRs are almost equally flexible with significant energy required to deform the structure (2.076201e−07 for TLR-3/vaccine complex and 2.267755e−07 for TLR-4/vaccine complex). The deformability graphs also demonstrated narrow peaks of stiffer regions with mostly flexible regions evidencing the stability of the construct. We can see from the elastic maps that atoms in the TLR-3 complex are not as stiff as the ones in the TLR-4 complex, with the findings being consistent with the co-variance plots showing swifter correlated motions (displayed in red) in the TLR-3 complex as compared to the TLR-4 complex. Nonetheless, the difference is minimal, indicating that both complexes are flexible when subjected to electric fields. The findings are summarized in Fig. 5 (for TLR-3/vaccine complex) and Fig. 6 (for TLR-4/vaccine complex).

## Discussion

*Naegleria fowleri* is a lethal water-borne microorganism associated with brain infection, also known as brain-eating amoeba. The number of deaths caused by *N. fowleri* infection has risen to 97%, and prominent incidents over the past 5 years have brought *N. fowleri* significant media coverage^34^. The public has kept a close eye on several such incidents. The clinical signs of primary amoebic meningoencephalitis are severe and almost often deadly, even though this disease seems uncommon compared to other infections^35^. There are a few antibiotics utilized as a medication against *Naegleria fowleri*, and one of the most reliable and used antibiotics is amphotericin B, evidenced by in vitro studies. Hygromycin, clarithromycin, roxithromycin, and zeocin are the additional drugs used to treat brain-eating bacterial infections^35^. A bioinformatic-based vaccination against *Naegleria fowleri* has been developed here to treat its infections effectively, using an approach previously published^36^. Disease-causing non-homologous proteins were examined, and out of the five protein sequences that made the final list, immune cell epitopes were designated based on their immunogenicity and allergenicity activities. Non-allergenic 10 B-cell, T-cell, and HTL epitopes were identified as viable targets for vaccine development, with antigenicity exceeding a threshold of > 0.5.

The main purpose of the vaccine design is to pave the way toward the ultimate cure for a long time, and T-cell epitopes play a vital role in this regard^37^, making them a crucial aspect of vaccine design. Predicted B-cell and T-cell epitopes were joined using different linkers like EAAAK, GPGPG, and AAY. These linkers were utilized based on similar studies^38–40^. Physiochemical analysis was done to predict the properties of the vaccine construct, along with antigenicity, allergenicity, and toxicity investigation, as reported in previous studies^41^. The RpfE adjuvant was used to enhance the immunogenicity and expression of the vaccine construct. RpfE is a TLR4 agonist that can be used as a natural adjuvant to increase the immunogenicity of a vaccine construct^42^. Using a TLR agonist as an adjuvant can increase the processing of antigens by antigen-presenting cells (APCs). It can help improve the potential of a vaccine in formulation with novel adjuvants, which can effectively impart superior immunity, as Naveed et al.^43^ reported. The use of RpfE as an adjuvant in vaccine design is intended to increase the immunogenicity of the vaccine by enhancing the immune response to the antigen. The response to RpfE is expected to be both local and systemic, as it activates innate immune responses and promotes the differentiation of Th1 and Th17 cells^44^. We used RpfE because compared to other adjuvants, RpfE has the advantage of being a protein, allowing its structure and function to be modified as necessary for optimal immunogenicity and minimal toxicity^45^. RpfE can be genetically fused to protein antigens, ensuring adjuvant-antigen co-delivery into the same cell and endocytic cargo^46^. It leads to more effective activation of innate and adaptive immune responses.

Molecular docking of the vaccine construct's 3-D structure was done with TLR-3 and TLR-4 receptors of the host, generating a significant immune response against the vaccine after the interaction. Molecular dynamic studies assessed the strength and reaction of the docked complex of the vaccine construct and TLRs in the host. Molecular dynamics simulation was also used to confirm the stable nature of the docked complex. Molecular docking and molecular dynamics (MD) simulations are computational techniques used to study the interactions between molecules, such as a vaccine candidate and its target receptor^47,48^. In the context of vaccine design, these techniques can predict a vaccine candidate's binding affinity and stability with immune receptors such as TLR-3 and TLR-4. By performing docking and MD simulations, researchers can gain insights into the molecular mechanisms of vaccine–receptor interactions and assess the potential efficacy of a vaccine candidate. For example, a study on a multi-epitope-based subunit vaccine against the West Nile virus used molecular docking to show that selected epitopes had a stronger binding affinity with human TLR-4^49^. Such analyses support the use of these TLRs in this study. Immune simulations through C-immsim exhibited substantial interpretations of immune response after the injection of the vaccine as an antigen in the host. These include the production of memory B and T-cells, stimulation of helper T-cells and cytotoxic T-cells, and the rise in the levels of interferon IFN-γ and interleukins IL-2, with proficient development of IgG and IgM antibodies after exposure to the vaccine. While C-ImmSim provides a framework for simulating the development of immunological memory^50^, it is important to note that the results of such simulations should be validated through experimental studies. In particular, it would be valuable to compare the results of C-ImmSim simulations with experimental data on the generation of immunological memory following immunization with synthetic peptides. Such comparisons could help to assess the accuracy and reliability of C-ImmSim in predicting the development of immunological memory. For expressional analysis, the *E. coli* K12 strain was utilized, and the codon was optimized up to the desired GC content of 60% and CAI of 0.9 and expressed in *E. coli*.

The vaccine design process aims to address the critical need for an effective treatment against *Naegleria fowleri* infections. Researchers hope to induce a robust and specific immune response in the host by targeting non-homologous proteins and selecting immunogenic, non-allergenic epitopes. However, the development of an *N. fowleri* vaccine also presents challenges. Clinical validation through preclinical and clinical studies is essential to ensure the vaccine's safety and efficacy in humans. For example, Gebre et al.^51^ observed rapid induction of antigen-specific binding and neutralizing antibodies in response to vaccine candidates. Their study highlighted the mRNA vaccine's ability to elicit early immune responses, showcasing increased levels of IL-5, IL-6, and MCP-1 cytokines post-immunization. Additionally, Erasmus et al.^52^ highlight potent immune responses triggered by single and prime/boost vaccination regimens in mice. These studies emphasize strong CD4+ and CD8+ T cell responses, enduring plasma and memory B cell reactions, and significant IgG antibody production after intramuscular vaccination. That opens new avenues for the proposed vaccine candidate against *N. fowleri.* If we use the same inoculation route as discussed in other research works and succeed in stimulating the mentioned immune cells, we have a real chance of success against brain-eating amoebae.

## Conclusions

The current investigation found that the vaccine design had excellent physicochemical properties and immunological responses to *Naegleria fowleri*. Using acknowledged immune-informatics techniques, it was discovered that this vaccination would induce an immunological response against *N. fowleri* in the host. Immune Simulation results like antibody production up to 7000 titers, interferon 400,000 ng/ml, and GRAVY index of − 0.349, showing the vaccination's immunological response supported our theory. The designed construct could be a suitable candidate for wet-lab studies against *N. fowleri*, employing a variety of serological tests to elicit the response.

## Data Availability

The data generated in this research work has been included in the manuscript.
